# Gastrointestinal Microbiome Dysbiosis in Cancer Development: Mechanisms and Biomarker Potential

**DOI:** 10.1002/mbo3.70404

**Published:** 2026-09-09

**Authors:** Negar Asgari, Mehrasa Asghari, Touraj Farazmandfar

**Affiliations:** ^1^ Institute of Regenerative Medicine and Biotherapy Université de Montpellier, CHU de Montpellier Montpellier France; ^2^ Medical Cellular and Molecular Research Center Biomedical Research Institute, Golestan University of Medical Sciences Gorgan Iran; ^3^ Department of Molecular Medicine Faculty of Advanced Medical Technologies, Golestan University of Medical Sciences Gorgan Iran

**Keywords:** carcinogenesis, dysbiosis, gastrointestinal cancer, microbiome

## Abstract

Increasing evidence indicates that alterations in the gastrointestinal microbiota are associated with the initiation and progression of gastrointestinal cancers. Abundant evidence suggests that gastrointestinal microbiota dysbiosis plays an important role in the development of gastrointestinal cancers by producing proinflammatory and immunosuppressive signals. This review examines the role of microbiome dysbiosis in the pathogenesis of gastrointestinal malignancies. The composition of the microbiota varies greatly among individuals and depends on various factors such as age, diet, lifestyle, genetics, medications, and environmental factors. Given this diversity, a comprehensive characterization of the gastrointestinal microbiota is crucial to elucidate its role in carcinogenesis. Therefore, investigating the microbial composition of the host gastrointestinal tract can provide important information about the status of gastrointestinal cancers.

## Graphical abstract

Dysbiosis of the gastrointestinal microbiome plays a role in carcinogenesis by inducing chronic inflammation, DNA damage, and activation of signaling pathways such as NF‐κB, STAT3, and Wnt/β‐catenin. Microorganisms such as *Helicobacter pylori*, Fusobacterium nucleatum, and Bacteroides fragilis promote the development of gastrointestinal tumors through their toxins and metabolites. Alterations in microbiome composition have great potential as noninvasive biomarkers for early detection of gastric, colorectal, and pancreatic cancers.

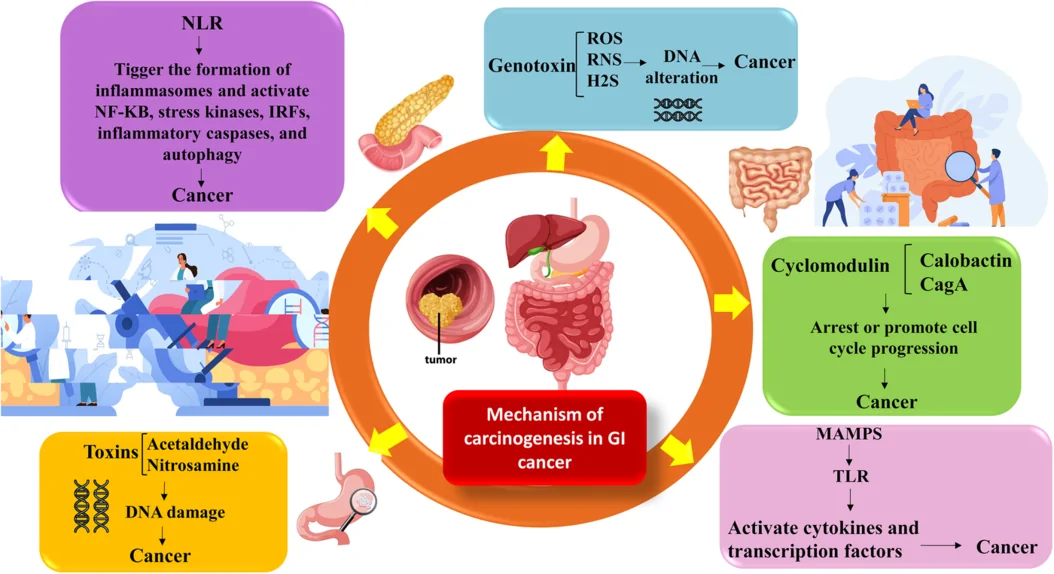

## 1 Introduction

Gastric cancer (GC) is one of the most common malignancies worldwide and remains a major cause of cancer‐related mortality. It affects more than one million people annually and accounts for approximately 5.7% of all cancers. The prognosis of the disease is poor, and a substantial proportion of patients are diagnosed at advanced stages, which contributes to poor clinical outcomes (Mamun et al. 2024). The development of GC is associated with several risk factors, such as *Helicobacter pylori* (*H. pylori*) infection, stomach ulcers, obesity, smoking, high salt diets, alcohol consumption, radiation exposure, Epstein‐Barr virus, and epigenetic and genetic factors (Duan et al. 2025). *H. pylori* is the major established infectious risk factor for gastric cancer and contributes substantially to gastric carcinogenesis. In recent years, new information has been revealed about the tactics used by *H. pylori* to survive in the acidic environment of the stomach, persistent infection, and host dysfunction (Piscione et al. 2021). Recent research highlights the potential role of non‐*Helicobacter pylori* microbes in promoting gastrointestinal tumorigenesis in murine models. Research indicates that proton pump inhibitors decrease stomach acid production, which can lead to an increase in bacterial growth (Shanika et al. 2023).

In addition to *H. pylori* infection, other host and environmental factors influencing the development of the disease, dysbiosis, or changes in the type and function of the normal microbiome of the stomach may also contribute to carcinogenesis. A dysbiotic gastric microbial community may develop due to chronic mucosal inflammation caused by the microbiome, which can change the gastric environment (Piscione et al. 2021). Continuous activation of the host's immune system by the gut microbiome leads to disordered interactions between host epithelial cells and microbes, which subsequently creates a chronic inflammatory state. The normal inhabitants of our gut become pathobionts when the natural symbiosis becomes dysbiotic for various reasons, and these pathobionts are likely to cause cancer in response to dietary and environmental carcinogens. The content of the gut microbiota and the occurrence of cancer are both significantly influenced by diet. Metabolic byproducts of microbial metabolism derived from food components control our health and disease states. Previous research has shown that cancerous tissues in the digestive tract have a more diverse and abundant microbiota than stromal tissue (Huang et al. 2023). Additionally, a study revealed that microbiological diversity is higher in the advanced stages of GC than in the early stages (Z. Wang et al. 2020).

This article reviews the role of gastrointestinal microbiome dysbiosis and its associated molecular mechanisms in the development and progression of gastric cancer.

## 2 Microbiome

**Figure 1:**
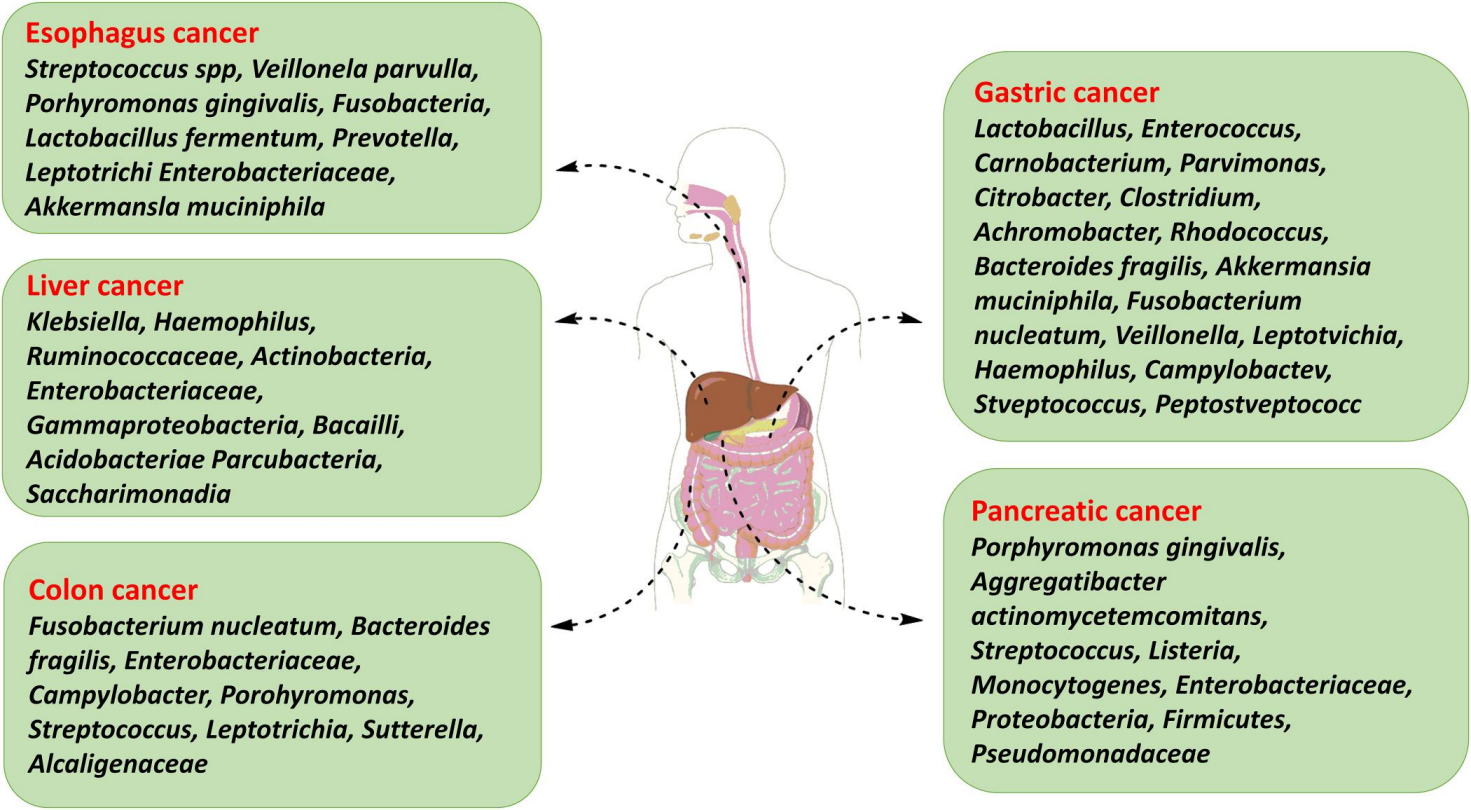
Overview of microbiota and cancers of the luminal GI tract.

It is estimated that about 38 trillion bacteria inhabit the human body. These bacteria can influence diverse physiological processes and disease susceptibility. The human microbiome includes bacteria, fungi, eukaryotic protozoa, and viruses. Typically, the microbiome functions as a meta‐organism and has a healthy symbiotic relationship with its host. It utilizes the host's nutrient‐rich environment and aids in the digestion and metabolism of these materials (Z. Wang et al. 2020). The microbiome of the mouth, skin, and gut has very rich and diverse populations of bacteria. In contrast, the vaginal microbiome has received less attention but is nonetheless well‐studied and known to contain particular and dominant microbial populations. However, the origins and colonization dynamics of these microbiomes remain under investigation. Microbial niches may influence cancer progression through interactions mediated by altered microbiome topologies, or direct interactions between members or secreted metabolites (Malard and Guisan 2023) (Figure 1).

### 2.1 Oral Microbiome and Gastrointestinal Cancers

The oral cavity is always exposed to microbes and contains about 500 to 700 bacterial species (Rajasekaran et al. 2024). The oral cavity is a unique ecological system, each sub‐sector of which is composed of distinct microbial communities. These sub‐sectors include the oral mucosa, palatal mucosa, dorsum of the tongue, saliva, soft surfaces that are being shed, and non‐shed tooth surfaces that are constantly exposed to bacterial plaque growth. Saliva and dental plaque have the highest bacterial diversity, while the oral mucosa microbiome has the lowest diversity (Morgan et al. 2022). Several systemic malignancies have been associated with specific oral microorganisms commonly present in the oral cavity. Dysbiosis of the oral microbiome is associated with pancreatic cancer, colorectal cancer (CRC), and inflammatory bowel disease. There are two types of relationships between oral bacteria and distant malignancies. In most cancers, oral microbes are not directly involved in tumor pathogenesis, but there is a persistent change in the composition of the oral microbiome associated with cancer that may be used as a potential biomarker in cancer prognosis (Vogtmann et al. 2020).

*Porphyromonas gingivalis*, one of the primary oral infections, makes oral cancer cells more aggressive and resistant to chemotherapy drugs. *P. gingivalis* assists tumor growth by breaking down dead precancerous cells and disabling myeloid‐derived suppressor cells. This bacterium stimulates the primary immune response through Toll‐like receptor 4 (TLR4), myeloid differentiation 88 (MyD88) factor, and MyD88‐independent pathways. This process activates the nuclear factor‐κB (NF‐κB) pathway and leads to the release of cytokines that cause inflammation (Wen et al. 2020). TLR4 is overexpressed in cancerous pancreas compared to healthy pancreas, which is an interesting marker in the development of pancreatic cancer. In addition, the lipopolysaccharide of this bacterium also causes the development of pancreatic cancer by blocking the MyD88‐dependent pathway (through dendritic cell‐mediated T helper 2 differentiation induction) (Yan et al. 2025).

Clinical studies have shown that tumor tissues in the digestive system contain more oral microbiota, such as *streptococci*, *Peptostreptococcus*, and *fusobacterium*, than adjacent non‐tumor tissues. In addition, molecular analysis of the gastrointestinal microbiome of gastric cancer patients using 16 s rRNA gene sequencing showed that oral bacteria such as *Parvimonas micra*, *S. anginosus*, *Slackia exigua*, and *Peptostreptococcus stomatis* are also present in these areas. The change in the acidic environment of the stomach likely allows oral bacteria to colonize there. Levels of the bacterium *Lactobacillus brevis* are also lower in gastric cancer tissue compared to non‐tumor tissue. In contrast, levels of other oral bacteria, such as *Streptococcus* and *Clostridium*, are also significantly higher in this comparison (Qian et al. 2024).

### 2.2 Gut Microbiome and Gastrointestinal Cancer

The colon contains the highest density of microorganisms in the gastrointestinal tract and represents the major reservoir of the human gut microbiota. The gut microbiome plays a key role in controlling the host immune system, both in the gut and throughout the body (Dupont et al. 2020). It contains the significant genus *Lactobacillus*, some species of which are used to treat several digestive disorders. The lactic acid produced by this bacterium leads to the modification of many important biological processes, including immune system modulation, anti‐inflammatory factors, and anti‐cancer pathways (Saberpour et al. 2025). In a case‐control study, GC patients had higher levels of P. copri and P. acnes in their stomachs than controls. These findings indicate that these species increase the risk of GC (Gunathilake et al. 2019). One of the most common bacterial genera in the human gut microbiome is *Protella*, which is resistant to host‐derived reactive oxygen species (ROS). This bacterium produces the redox protein thioredoxin as part of its inflammatory activity in several diseases (Kunst et al. 2023).

### 2.3 Gut Microbiota and Immune Imbalance in Cancer

The homeostasis of the gut microbiome is regulated by the host's immunity, and in turn, the microbiome shapes the behavior of the immune system in the gut. Gut microbiota can influence not only gut immunity but also immune responses at other mucosal sites through the circulation (Hafezi et al. 2015; Taghizadeh et al. 2014; Rafiei 2013). Given that the gastrointestinal tract is frequently exposed to external pathogens, the mucosal immune system is the primary defense against invasion. Intestinal epithelial cells, acting as immune barriers, commonly show pattern recognition receptors such as nodule‐like receptors (NLRs) and TLRs. Upon contact with microorganisms, these receptors recognize flagellins, peptidoglycans, lipopolysaccharides, and cell wall lipoproteins (Amemiya et al. 2021).

Cancer immunoediting is commonly described as a dynamic process comprising three phases: elimination, equilibrium, and immune escape. Elimination is the process by which the host immune system recognizes and destroys tumor cells. Equilibrium is a dynamic state in which cancer cells cannot be completely eradicated or divide rapidly because they are still under the control of the immune system. The final stage involves immune escape, in which tumor cells grow despite the surveillance of the host immune system (Zhou et al. 2021).

Recent findings suggest that the effects of the gut microbiota on colorectal cancer are not limited to regulating the immune response, but also influence tumor behavior through reprogramming metabolic, neural, and endocrine axes. Microbial metabolites are able to simultaneously regulate signaling pathways related to cell proliferation, angiogenesis, inflammation, and immune cell function. These multiaxial interactions suggest that the microbiome acts as a systemic regulator and can influence the initiation, progression, and even response to treatment of gastrointestinal cancers (Bautista et al. 2026).

### 2.4 Blood Microbiome and Gastrointestinal Cancer

Blood was considered sterile in non‐infectious conditions. Although microbial DNA has been identified in blood using culture‐independent sequencing approaches, the existence of a stable microbiome residing in blood remains controversial. Because blood is an environment with low biomass, contamination from reagents, laboratory environments, and other body sites poses a major methodological concern. One study indicated that microorganisms that don't grow in blood cultures typically originate from the mouth or gut and are often inactive. It has also been found that changes in the blood microbiota are associated with several non‐pathogenic diseases (Khan et al. 2025). Previous studies have shown that the composition of the blood microbiome of patients with gastric cancer is significantly different from that of the healthy group. These studies also showed a significant correlation between the blood microbiome and tumor size, invasion intensity, and metastasis in gastric cancer. In that study, patients with stomach cancer had a higher concentration of three bacterial species, namely, *Bacteroides*, *Haemophilus parainfluenzae*, and *Acinetobacter*. Furthermore, lymphatic and non‐lymphatic metastases of gastric cancer have different serum microbiota compositions (Dong et al. 2019). Another study showed that the composition of the basal blood microbiota in patients with advanced CRC who responded to immunochemotherapy (oxaliplatin + capecitabine + adaptive T‐cell immunotherapy) was significantly different compared to patients who did not respond. Their results showed that responding patients had higher concentrations of the genera *Bifidobacterium*, *Lactobacillus*, and *Enterococcus* (Yang et al. 2021).

### 2.5 Microbiome Dysbiosis in Gastrointestinal Cancer

During the early stages of GC, *Ralstonia*, *Sphingobium*, *Ochrobactrum*, *Anoxybacillus*, and *Pseudoxanthomonas* species tend to be found in abundance. As the stages advance, *Tsukamurella*, *Burkholderia*, and *Salinivibrio* species become more prevalent (X. Wang et al. 2020). A recent study has elucidated the microbial composition of different gastric cancer subtypes. Their results showed that *Proteobacteria* and *Acidobacteria* were prevalent in adenocarcinoma samples, while *Fusobacteria* and *Bacteroidetes* were prevalent in signet ring cell carcinomas (Ravegnini et al. 2020).

### 2.6 Gut Barrier Dysfunction and Microbial Translocation

The intestinal mucosal barrier is one of the most important defenses against the entry of microorganisms and microbial products into the bloodstream. This barrier is composed of epithelial cells, tight junctions, the mucosal layer, and the mucosal immune system, and plays a fundamental role in maintaining intestinal homeostasis. Recent evidence suggests that microbial dysbiosis, especially in aging, can disrupt the function of this barrier by reducing microbial diversity, weakening intercellular junctions, and increasing epithelial permeability (leaky gut). As a result, lipopolysaccharides, peptidoglycans, and other bacterial products are transported to the lamina propria and the bloodstream, and through the activation of pattern recognition receptors such as TLRs and NLRs, they induce the production of inflammatory cytokines and chronic systemic inflammation. This persistent inflammation, together with increased oxidative stress, DNA damage, and an altered tumor microenvironment, provides favorable conditions for the initiation, progression, and metastasis of gastrointestinal cancers. On the other hand, intestinal barrier dysfunction can also impair antitumor immune responses and reduce the efficacy of anticancer therapies, especially immunotherapy. Therefore, maintaining the integrity of the mucosal barrier and restoring the balance of microbiota have been considered an emerging strategy in the prevention and treatment of gastrointestinal cancers (Liang et al. 2026).

## 3 Mechanisms of Microbiome Carcinogenesis

Microbiome dysbiosis simultaneously utilizes multiple mechanisms to accelerate tumor growth in the host. Bacterial strains that thrive in a neoplastic environment may exploit these mechanisms to promote cancer metastasis and relapse during chemotherapy. Bacterial infections represent one of the principal mechanisms through which dysbiosis contributes to tumor development. Bacteria produce large amounts of ROS and toxins that disrupt cellular regulatory processes, the cell cycle, and the production of inflammatory mediators, which can lead to tumor formation. These bacterial toxins can induce mutations and genotoxic stress by targeting critical cellular pathways. Cyclomodulins are bacterial toxins that alter the cell cycle and render bacterial strains that express them highly carcinogenic. Some human cancers have been linked to bacterial strains that produce cyclomodulins, such as *Escherichia coli*, which produces colibactin, and *H. pylori* strains that produce cytotoxin‐associated gene A (CagA). Another mechanism of carcinogenesis by bacteria is activation of microorganism‐associated molecular patterns through translocation. These factors activate TLRs, which subsequently activate cytokines and transcription factors, which cause an immune imbalance. This immune imbalance activates multiple signaling pathways mediated by NLRs that induce inflammation or activation of NF‐κB, stress kinases, interferon regulatory factors, and inflammatory caspases, ultimately leading to the clearance of genotoxins or damaged cells by autophagy. The output of bacterial metabolic activities can also lead to the activation of toxic substances such as nitrosamines and acetaldehyde, which have genotoxic effects and lead to carcinogenesis. In addition, reactive nitrogen species, ROS, and hydrogen sulfide, which are released by some bacteria, can negatively affect antitumor control pathways (Ionescu et al. 2025).

Recent reviews suggest that carcinogenesis caused by microbiome dysbiosis is not the result of a single mechanism, but rather the result of a simultaneous interaction of inflammatory, immune, metabolic, and genotoxic pathways. Among them, bacterial metabolites, alterations in bile acid metabolism, disruption of mucosal barrier function, regulation of innate and adaptive immune responses, and activation of signaling pathways such as NF‐κB, STAT3, and Wnt/β‐catenin act in a networked manner to create a microenvironment conducive to tumor initiation, progression, and metastasis. This perspective suggests that functional assessment of the microbiome, in addition to examining microbial composition, is essential for a complete understanding of carcinogenesis (Ağagündüz et al. 2023).

Recent studies have shown that in addition to producing carcinogenic metabolites, the gut microbiota also plays a role in the metabolism of many environmental chemicals and food compounds and can increase or decrease the toxicity or carcinogenicity of these compounds. Bacterial enzymes alter the metabolic pathways of carcinogens, altering the extent of epithelial cell contact with active metabolites, thereby affecting the severity of DNA damage, oxidative stress, and inflammatory responses. This highlights the importance of the metabolic function of the microbiome in addition to its bacterial composition (Pan et al. 2025).

**Figure 2:**
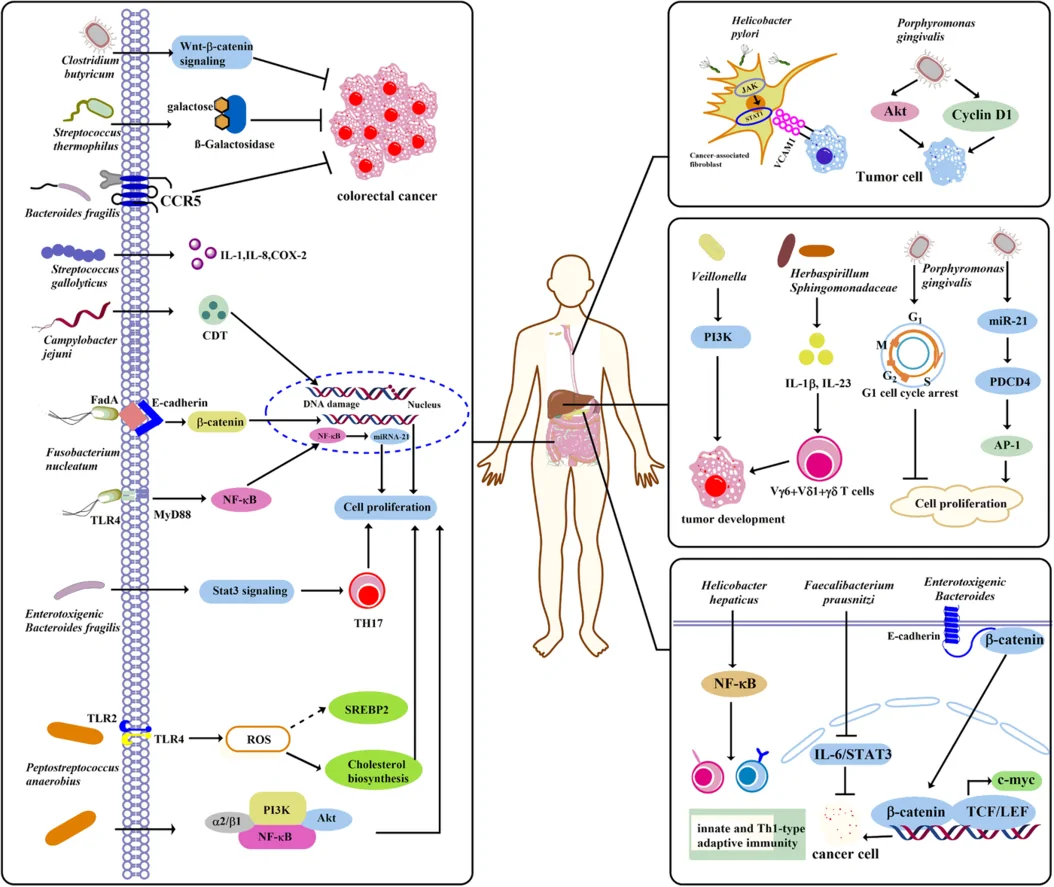
A review of the mechanisms of tumor progression by microbiome bacteria. Pathogenic bacteria commonly originate from the oral and gastrointestinal flora. This flora can induce host immune responses and lead to cell cycle arrest, DNA transcriptional changes, DNA damage, accumulation of reactive oxygen species, and activation of multiple signaling pathways such as STAT3, NF‐κB, and Wnt/β‐catenin, ultimately leading to tumor initiation, progression, and metastasis.

Microbial diversity can play an indirect role in cancer development by causing systemic diseases or mucosal inflammation. Most studies in this area are related to the oral and intestinal microbiome (Figure 2). *H. pylori* increases the expression of vascular cell adhesion protein 1 (VCAM1) in tumor‐associated fibroblasts through the Janus kinase‐activator of transcription (JAK‐STAT) signaling pathway in gastric cancer. In support of these results, some studies show that VCAM1 levels are positively correlated with tumor progression and poor prognosis in gastric cancer patients. Additionally, the interaction between cancer‐associated fibroblast‐derived VCAM1 and integrin αvβ1/5 results in increased invasion of gastric cancer cells, both in vitro and in vivo (Shen et al. 2020).

*Fusobacterium nucleatum* (*F. nucleatum*) is a commensal bacterium in the human oral cavity. FadA adhesion complexes from *F. nucleatum* have previously been shown to be associated with cancer‐promoting transcriptional changes in CRC cell lines. Experimental studies have suggested that *F. nucleatum* may promote CRC cell proliferation and migration through TLR4/MyD88/NF‐κB‐associated signaling, including modulation of miR‐21 expression (Dewan et al. 2025). In mouse models, colonization with *Enterotoxigenic Bacteroides fragilis* has been shown to promote intestinal tumorigenesis through inflammatory and STAT3/Th17‐associated mechanisms (Allen et al. 2022). Another reason for the carcinogenicity of the microbiome may be the higher abundance of anaerobic *Peptostreptococci* in stool samples from patients with CRC compared to the healthy population. This bacterium can bind to α2/β1 integrin through its surface protein, putative cell wall binding repeat 2. This binding leads to the stimulation of focal adhesion kinase phosphorylation and activation of the PI3K/AKT pathway in CRC cells. In addition, the activities of this bacterium lead to increased cell proliferation and activation of the NF‐κB signaling pathway, which ultimately leads to increased inflammation and tumor progression (Gopalakrishnan et al. 2025). Some of the microbiota associated with cancer are presented in Table 1.

**Table 1:** A history of the relationship between the gut microbiome and various types of gastrointestinal cancers.

Type of gastrointestinal cancer	Type of microbes	Mechanism	References
Esophagus cancer	Gut microbiome	Changes in expression profile, increased proliferation of gastric cardia progenitor cells, and modulation of acute and chronic immune responses.	(Polyzos et al. (2018))
		Dysplasia induction due to alteration of the gut microbiome and esophageal microenvironment by high‐fat diets, which leads to inflammation and proliferation of stem cells.	(Nardone et al. (2017))
Gastric cancer	*Helicobacter pylori*	Induction of DNA double‐strand breaks in epithelial cells	(Hartung et al. (2015))
		Alteration of the gastric microbiota, which is associated with bacterial diversity and overgrowth.	(Sethi et al. (2018))
Colon cancer	*Escherichia coli*	It leads to colon cancer through p38 MAPK, which induces COX‐2 expression in human macrophages.	(Castaño‐Rodríguez et al. (2017))
		Acceleration of cell cycle arrest due to inflammatory reactions, DNA damage, secretion of pathogenic factors, activation of COX‐2, etc. They also disrupt the intestinal vascular barrier.	(Dalal et al. (2021))
	*Fusobacterium nucleatum*	The conversion of TLR4 signaling to MYD88 by this bacterium leads to the stimulation of nuclear factor B and increased production of miR‐21, which in turn can cause malignancies.	(Ferreira et al. (2018))
		Induction of Annexin A1 production in malignant cells via E‐cadherin and FadA factors.	(Rubinstein et al. (2019))
	*Campylobacter jejuni*	The lethal toxin produced by this bacterium is genotoxic and induces CRC. Exogenous p‐cresol significantly increases DNA damage in HT29 and Caco‐2 cell lines, causing DNA damage and altering cell cycle kinetics at high doses.	(Y. Zhang et al. (2018))
	*Bacteroides fragilis*	Interaction with IL‐17 and activation of NF‐κB.	(Chung et al. (2018))
	*Streptococcus gallolyticus*	Activation of NF‐κB and IL‐8 promotes CRC cell proliferation through β‐catenin signaling.	(Kumar et al. (2017))
Liver cancer	Gram‐positive gut bacteria	These bacteria produce lipoteichoic acid and deoxycholic acid, which, in combination, lead to increased expression of COX2 and SASP factors in DCA‐induced senescent hepatic stellate cells.	(Li et al. (2019))
	SCFA‐producing bacteria	Intestinal bacteria ferment soluble dietary fibers into SCFA, causing fibrosis and proliferation of liver cells and inducing bile duct cancer.	(Singh et al. (2018))
	Gut microbiome	Dysbiosis of the gut microbiota leads to the secretion of IL‐25 and subsequent secondary activation of macrophages in the tumor microenvironment and their secretion of CXCL10, and ultimately the development of HCC.	(He et al. (2019))
Pancreatic cancer	*Bifidobacterium pseudolongum*	Facilitates differentiation of macrophages into M1.	(Pushalkar et al. (2018))
	Gut microbiome	The gut microbiome is altered by a high‐fat diet, increasing inflammatory stimulation and proliferation of dysplastic stem cells.	(Xu et al. (2021))

Abbreviations: COX‐2, Cyclooxygenase‐2; CRC, Colorectal cancer; CXCL, C‐X‐C motif chemokine ligand; DCA, Dichloroacetate; HCC, Hepatocellular carcinoma; IL, Interleukin; MAPK, Mitogen‐activated protein kinases; SASP, Senescence‐associated secretory phenotype; SCFA, Short‐chain fatty acids.

## 4 The Development of Gastric Cancer Is Induced by *H. pylori*

**Figure 3:**
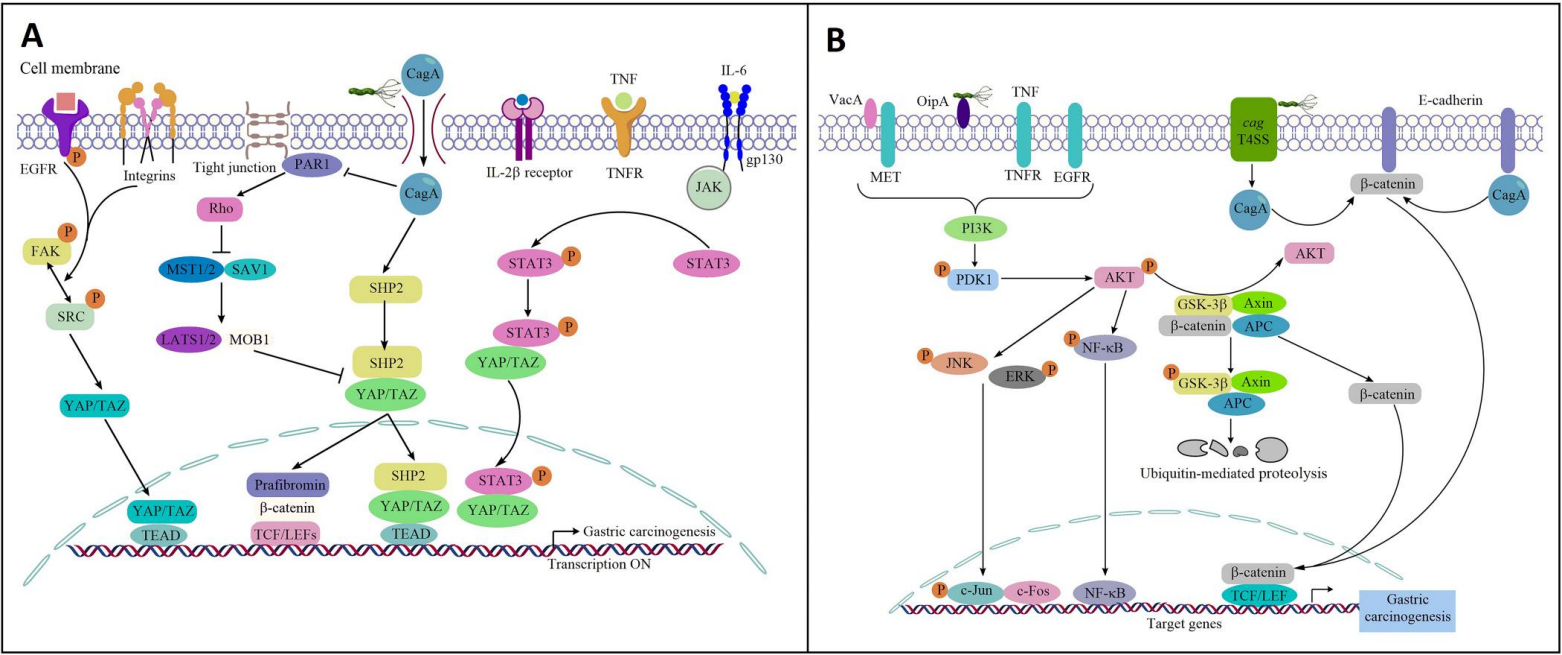
*Helicobacter pylori* stimulates the development of gastric cancer. (A) Inflammatory cytokines produced by this bacterium can induce the STAT3, Hippo/YAP, and Wnt/β‐catenin pathways and promote gastric tumorigenesis. Infection with this bacterium causes the activation of the EGFR and FAK/SRC/YAP pathways. Infection with this bacterium can also disrupt tight junctions and stimulate the intracellular factor SHP2, leading to the activation of the Wnt/β‐catenin and Hippo/YAP signaling pathways. (B) After *H. pylori* binding, CagA, VacA, OpiA, and TNF signals activate the Akt pathway, which then activates the JNK/ERK, Wnt/β‐catenin, and NF‐κB pathways. In addition, CagA can also directly lead to the activation of the Wnt/β‐catenin pathway.

*H. pylori* infection has been shown to activate STAT3 signaling through multiple mechanisms. It can persistently activate STAT3 by increasing the expression of interleukin 6 (IL‐6). Furthermore, the pathogenicity factor CagA of *H. pylori*, when delivered to host epithelial cells, leads to activation of STAT3 via the SHP‐2 pathway and induction of transcription of STAT3 target genes. *H. pylori* also interacts with TLR2 and increases inflammatory responses and STAT3 activation (Raza et al. 2025). Duan et al. showed that *H. pylori*, through the SRC‐YAP1 (Yes‐associated protein 1) pathway, leads to overexpression of DAB2 under the induction of STAT3 and enhances gastric carcinogenesis (Duan et al. 2024). Notably, *H. pylori* infection can activate fibroblast growth factor receptor 4 through the STAT3 signaling pathway, a process that is associated with gastric cancer development (X. Zhang et al. 2022). It has also been shown that *H. pylori* can downregulate other components of the JAK‐STAT pathway. This is likely used as a strategy to evade the immune system (Xi et al. 2023) (Figure 3A).

*H. pylori* triggers the NF‐κB signaling pathway through multiple mechanisms. Specifically, CagA directly stimulates NF‐κB and increases its translocation between the nucleus and cytoplasm. *H. pylori* activates the IkappaB kinase complex, which then activates the NF‐κB pathway by boosting the production of inflammatory mediators like tumor necrosis factor (TNF)‐α and IL‐1β (M. Chen et al. 2022). Chen et al. showed that *H. pylori* can induce the PIEZO1/YAP1/CTGF pathway through the NF‐κB signal and cause the transformation of the GC microenvironment to induce gastric cancer (B. Chen et al. 2023). *H. pylori* infection can also stimulate the expression of RAS‐like activator protein 2 via the NF‐κB pathway and increase gastric carcinogenesis via the β‐catenin pathway (Cao et al. 2022). Several lines of evidence indicate that sustained activity of the Wnt/β‐catenin pathway is significantly associated with the incidence, progression, and invasion of gastric cancer (J. Liu et al. 2022). The Wnt/β‐catenin pathway plays a key role in the development of early‐stage gastric cancer caused by *H. pylori*. It has been shown that this bacterium can disrupt the Wnt‐dependent cell polarity mechanism, leading to enteroendocrine differentiation and proliferation of stem cells (Takahashi‐Kanemitsu et al. 2023). CagA activates this pathway and increases the accumulation and nuclear translocation of β‐catenin. Recent evidence suggests that *H pylori*‐induced LRP8 expression facilitates the nuclear accumulation of β‐catenin and may play a role in gastric cancer progression (B. Liu et al. 2025). Nuclear accumulation of β‐catenin stimulates the expression of epithelial‐to‐mesenchymal transition transcription factors and confers stem cell‐like capabilities to tumor cells (Tang et al. 2020). Activation of this pathway also stimulates tumor cell invasion and is one of the hallmarks of cancer development in the stomach (Peng et al. 2021). Three major MAPK pathways, including the JNK, ERK, and p38 pathways, are modulated by *H. pylori* infection and play a pivotal role in the development of gastric cancer (Sharafutdinov et al. 2021) (Figure 3B).

## 5 Microbiota as a Biomarker in Gastrointestinal Cancer

Gastrointestinal cancer includes malignancies of the esophagus, stomach, colon, and rectum, although the most common types of gastrointestinal cancer worldwide are colorectal, pancreatic, and gastric cancers (Nasr et al. 2020). Despite rapid technological advances, the diagnosis and prognosis of GC are still in their infancy. A common challenge among gastrointestinal malignancies is the high morbidity and mortality associated with late‐stage diagnosis. Gastrointestinal cancers usually progress slowly before they affect a patient's digestive health (Castaño‐Rodríguez et al. 2017). Therefore, early detection is key to managing the disease and preventing it from getting worse. Finding and developing noninvasive biomarkers for early detection could be a real help.

Analysis of 16S rDNA through hierarchical clustering indicates the presence of two distinct microbial groups within the esophagus. The first type is mainly composed of *Streptococci*. The second type includes *Actinomyces*, *Protella*, *Veillonella*, *Neisseria*, *Fusobacterium*, *Haemophilus*, *Campylobacter*, *Rutia*, and *Porphyromonas*. This type, called meibomian, is absent from a healthy esophagus (Park and Lee 2020). Furthermore, based on research, it is hypothesized that a shift in the microbiota from normal to an increase of severalfold in the concentration of *Clostridiales* spp., *F. nucleatum*, *Erysipelotrichales*, or *P. gingivalis* could be applied as a prognostic factor for Esophageal squamous cell carcinoma (Yano et al. 2021).

In addition, due to the disruption of gastric acid secretion by *H. pylori*, other bacteria also accumulate in the stomach. Previous studies have shown that patients with gastric cancer have high levels of the genera *Lactobacillus*, *Lactococcus*, and the family *Lachnospiraceae*. Patients with gastric cancer have high densities of specific oral microbiota, such as *Haemophilus*, *Villonella*, *Fusobacterium*, *Leptotrichia*, and *Campylobacter*. This suggests that dysbiosis could be a useful diagnostic tool in gastric cancer. Studies show that, in addition to *Helicobacter pylori*, other bacterial species such as *Firmicutes*, *Proteobacteria*, *Actinobacteria*, and *Fusobacteria* have the potential to be used as biomarkers of gastric cancer (Janbabai et al. 2015; M. Wang et al. 2023).

Several studies have shown that the lack of microbiota in patients with CRC can increase the incidence of this type of cancer. They also reported that *F. nucleatum*, *Solubacterium morei*, *Parvimonas micra*, and *Peptostreptococcus stomatis* are associated with CRC (Ionescu et al. 2025). Several findings have also shown that the symbiotic oral anaerobic bacterium *F. nucleatum* is associated with CRC progression and metastasis. These results suggest that *F. nucleatum* has the potential to be a novel non‐invasive fecal biomarker for early detection of CRC (S. Wang et al. 2021). The abundance of *Streptococcus mitis* and *Neisseria elongata* in the salivary microbiome of pancreatic cancer patients was significantly lower compared to the healthy population. The researchers also observed that the ratio of *Leptotrichia* to *Porphyromonas* was significantly higher in the saliva of pancreatic cancer patients compared to the healthy population or people with other diseases. One study found that *F. nucleatum* and *P. gingivalis* were associated with an increased risk of pancreatic cancer (Tavanaeian et al. 2025).

## 6 Conclusion

Available evidence suggests that dysbiosis of the gastrointestinal microbiome may play a role in the development and progression of gastrointestinal cancers. Alterations in the composition and function of microbial communities may affect tumorigenesis by inducing chronic inflammation, producing metabolites and genotoxic substances, disrupting the epithelial barrier, modulating immune responses, and activating oncogenic signaling pathways. Among microbial agents, *Helicobacter pylori* and *Fusobacterium nucleatum* are important examples for which there is more experimental evidence of their association with carcinogenic processes. On the other hand, changes in the oral, gastric, and intestinal microbiomes, and even patterns of microbial DNA detectable in the bloodstream, have potential for use as diagnostic or prognostic biomarkers; however, the available evidence is still insufficient for routine clinical application of these markers.

Individual differences, diet, medications, geography, sampling method, and sequencing are among the most important limitations of current studies. Overall, a detailed understanding of the interactions between the microbiome, epithelial cells, the immune system, and the tumor microenvironment could lead to the identification of novel targets for the prevention, early detection, and treatment of gastrointestinal cancers. Despite significant progress, future studies should validate microbial markers independently and in combination using longitudinal and multicenter studies, standardized sampling and analysis methods, and multiomic approaches. Such an approach could lead to the development of microbiome‐based biomarkers and therapeutic strategies based on their modulation.

## Author Contributions

**Negar Asgari:** data curation, writing – review and editing, writing – original draft, investigation. **Mehrasa Asghari:** Writing – original draft, writing – review and editing, data curation. **Touraj Farazmandfar:** conceptualization, writing – original draft, writing – review and editing, validation, investigation, visualization.

## Funding

The authors have nothing to report.

## Ethics Statement

The authors have nothing to report.

## Consent

The authors have nothing to report.

## Conflicts of Interest

None declared.

## Data Availability

The authors have nothing to report.
